# Causal Effect of Relative Carbohydrate Intake on Hypertension through Psychological Well-Being and Adiposity: A Mendelian Randomization Study

**DOI:** 10.3390/nu15224817

**Published:** 2023-11-17

**Authors:** Chaojie Ye, Lijie Kong, Yiying Wang, Chun Dou, Min Xu, Jie Zheng, Ruizhi Zheng, Yu Xu, Mian Li, Zhiyun Zhao, Jieli Lu, Yuhong Chen, Weiqing Wang, Yufang Bi, Tiange Wang, Guang Ning

**Affiliations:** 1 Department of Endocrine and Metabolic Diseases, Shanghai Institute of Endocrine and Metabolic Diseases, Ruijin Hospital, Shanghai Jiao Tong University School of Medicine, Shanghai 200025, China; 2 Shanghai National Clinical Research Center for Metabolic Diseases, Key Laboratory for Endocrine and Metabolic Diseases of the National Health Commission of the PR China, Shanghai Key Laboratory for Endocrine Tumor, Ruijin Hospital, Shanghai Jiao Tong University School of Medicine, Shanghai 200025, China

**Keywords:** relative carbohydrate intake, hypertension, psychological well-being, adiposity, mediation, Mendelian randomization

## Abstract

Observations of the association between carbohydrate intake and hypertension are inconsistent, with mediating pathways unclear. We aimed to investigate the causal effect of relative carbohydrate intake on hypertension and the mediating roles of psychological well-being and adiposity. Using summary-level statistics of genome-wide association studies of European ancestry, we conducted univariable and multivariable Mendelian randomization (MR) to estimate the bidirectional causal association between relative carbohydrate intake (total energy-adjusted, mean: 42–51%) and hypertension (FinnGen: 42,857 cases/162,837 controls; UK Biobank: 77,723 cases/330,366 controls) and two-step MR to assess the mediating effects of psychological well-being indicators and adiposity traits on the association. MR estimates of hypertension from FinnGen and UK Biobank were meta-analyzed using the fixed-effect model given no heterogeneity. Meta-analyses of multivariable MR estimates from FinnGen and UK Biobank indicated that each one-SD higher relative carbohydrate intake was associated with 71% (odds ratio: 0.29; 95% confidence interval: 0.11–0.79) lower risk of hypertension, independently of other dietary macronutrients. Hypertension showed no reverse effect on carbohydrate intake. Five psychological well-being indicators and four adiposity traits causally mediated the association between relative carbohydrate intake and hypertension, including body mass index (mediation proportion: 51.37%), waist circumference (40.54%), waist-to-hip ratio (35.00%), hip circumference (24.77%), major depressive disorder (23.37%), positive affect (17.08%), depressive symptoms (16.52%), life satisfaction (16.05%), and neuroticism (11.22%). Higher relative carbohydrate intake was causally associated with lower hypertension risk, substantially mediated by better psychological well-being and less adiposity. Our findings inform causal targets and pathways for the prevention and intervention of hypertension.

## 1. Introduction

Carbohydrate intake accounts for approximately half or more of human daily caloric intake and can be potentially modified with intention [1]. Recently, the association between dietary intake of carbohydrates and cardiovascular health has received increasing attention [2]. Hypertension has become a major public health threat worldwide due to its rising prevalence and substantial impact on cardiovascular morbidity and mortality [3,4]. Current evidence regarding the association between carbohydrate intake and hypertension or cardiovascular disease seems controversial [1,5,6,7]. A Chinese nationwide cohort study has indicated a U-shaped association between carbohydrate intake and hypertension [5], whereas a network meta-analysis of randomized trials showed moderate-certainty evidence that compared with a usual diet, a low carbohydrate diet had beneficial effects on blood pressure reduction and weight loss in overweight or obese adults, but these effects largely disappeared at 12 months follow-up [6]. Meanwhile, findings from the Prospective Urban Rural Epidemiology study and the UK Biobank yielded no association between carbohydrate intake and cardiovascular disease [1,7]. These inconsistent results may be partly due to reverse causality, for example, high carbohydrate intake is common in individuals predisposed to hypertension, and residual confounding factors, such as other dietary macronutrients and total energy intake [1,5,7]. Moreover, the paucity of long-term randomized trials limits our knowledge of the permanent impact of carbohydrate intake on hypertension.

Thus far, whether carbohydrate intake causally associates with hypertension and whether hypertension has a reverse effect on carbohydrate intake remain unclear. Mendelian randomization (MR) is an alternative approach to infer the causality of lifetime risk factors with diseases by applying genetic variants fixed at conception and naturally randomly assigned to individuals as a proxy for the exposure, and thus to a large content to circumvent confounding or reverse causality [8]. Recent MR studies have suggested causal relationships of carbohydrate intake with depression and adiposity [9,10], while psychological health and adiposity have been recommended as risk factors for hypertension or cardiovascular disease [11,12]. These findings have inspired a novel hypothesis that carbohydrate intake may influence the development of hypertension, possibly through the mediating pathways of psychological health and adiposity.

To fill the knowledge gap, in this two-step, two-sample MR study, we investigated the bidirectional association between relative carbohydrate intake and hypertension and assessed the mediating effects of psychological well-being and adiposity in the association pathway, to facilitate prevention and intervention efforts to alleviate the burden of hypertension.

## 2. Materials and Methods

### 2.1. Study Design

**Figure 1:**
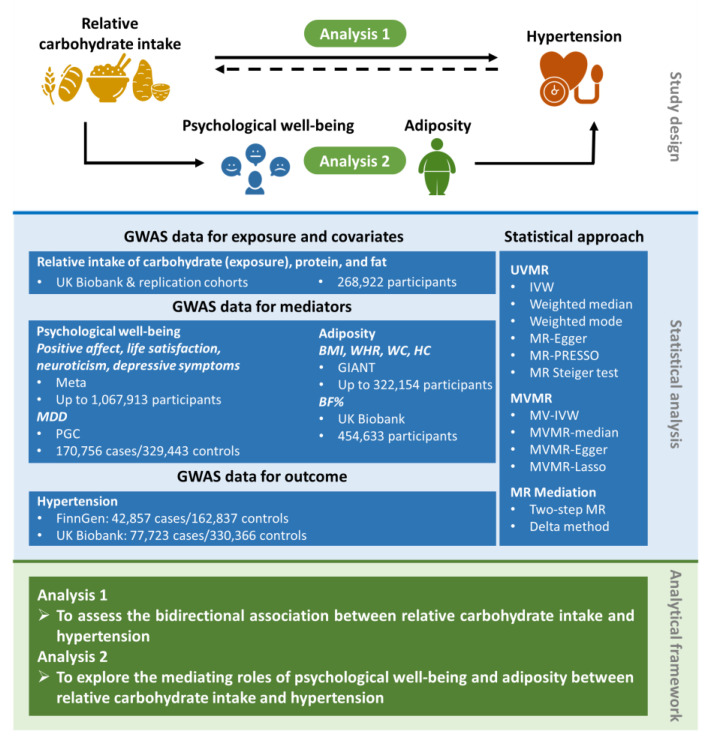
Overview of the MR study design. This MR study comprised two analysis phases. In Analysis 1, we assessed the bidirectional causal association between relative carbohydrate intake and hypertension by applying UVMR and MVMR. In Analysis 2, we performed the two-step MR to evaluate the mediating effects of psychological well-being indicators and adiposity traits in the causal association between relative carbohydrate intake and hypertension. BF%, body fat percentage; BMI, body mass index; GIANT, Genetic Investigation of ANthropometric Traits; GWAS, genome-wide association study; HC, hip circumference; IVW, inverse-variance weighted; MDD, major depressive disorder; MR, Mendelian randomization; MV-IVW, multivariable inverse-variance weighted; MVMR, multivariable Mendelian randomization; PGC, Psychiatric Genomic Consortium; PRESSO, pleiotropy residual sum and outlier; UVMR, univariable Mendelian randomization; WC, waist circumference; WHR, waist-to-hip ratio.

This MR study encompassed two analysis phases (Figure 1). We first performed univariable MR (UVMR) and multivariable MR (MVMR) to assess the bidirectional association between relative carbohydrate intake and hypertension. We then applied a two-step MR to explore whether and to what extent psychological well-being indicators and adiposity traits may play a causal role in the mediating pathway between relative carbohydrate intake and hypertension. The flow chart for the MR study is depicted in Supplementary Figure S1. This MR study is reported according to the Strengthening the Reporting of Observational Studies in Epidemiology Using Mendelian Randomization guidelines [13].

### 2.2. Data Sources for the Exposure, Covariates, Mediators, and Outcome

All the data used in the analyses were derived from genome-wide association studies (GWASs) conducted in European-descent participants from large-scale consortia or studies (Table 1). Information on ethical approval and participant informed consent for the GWASs can be found in the corresponding GWAS publications cited in the manuscript.

#### 2.2.1. Exposure and Covariates

GWAS data for macronutrient intake were obtained from the Social Science Genetic Association Consortium (SSGAC) of 268,922 participants [14]. The macronutrient intake data were collected as habitual or previous-day intake of the three macronutrients obtained through the comprehensive food-item questionnaires [14]. The relative intake of carbohydrate, protein, and fat was defined as the energy from each respective macronutrient divided by total energy intake, following the calculation formula: relative macronutrient intake = energy from macronutrient/(total energy^β^), where β is a correction factor to mitigate the bias caused by the non-linear association between relative macronutrient intake and total energy intake [14]. The mean value of relative carbohydrate intake (i.e., percentage of total energy intake from carbohydrates) ranged from 42% to 51% [14].

When selecting genetic instrumental variables (IVs), we screened for each macronutrient-related single nucleotide polymorphism (SNP) in the PhenoScanner database to assess any previous associations (*p* < 5.00 × 10^−8^) with plausible confounders (i.e., alcohol consumption, smoking status, physical activity, and education) in accordance with previous MR studies [9,10,19,20], and we excluded nine SNPs associated with certain confounders (for details, see Supplementary Table S1). Eventually, seven, four, and three SNPs at a genome-wide significant level (*p* < 5.00 × 10^−8^) and independent of each other (linkage disequilibrium [LD] r^2^ < 0.001 within 10,000 kb) were selected as the IVs for relative carbohydrate intake, protein intake, and fat intake, respectively (Supplementary Table S2).

#### 2.2.2. Mediators

We selected five psychological well-being indicators and five adiposity traits as potential mediators. GWAS data for positive affect, life satisfaction, neuroticism, and depressive symptoms were from a model-averaging genome-wide association meta-analysis of up to 1,295,946 participants from the SSGAC, Understanding Society, UK Biobank, 23 and Me, and Cohorts for Heart and Aging Research in Genomic Epidemiology Consortium [15]. GWAS data for major depressive disorder (MDD) were from the Psychiatric Genomic Consortium (*n* = 500,199), where individuals having received a clinical diagnosis or treatment for depression were classified as cases [16]. GWAS data for body mass index (BMI), waist-to-hip ratio (WHR), waist circumference (WC), and hip circumference (HC) were obtained from the Genetic Investigation of ANthropometric Traits consortium based on up to 322,154 participants aged 12 to 113 years [17,18]. GWAS data for body fat percentage (BF%) were from the UK Biobank (*n* = 454,633) [21].

In UVMR analyses, the IVs for each mediator were at a genome-wide significant level (*p* < 5.00 × 10^−8^) independently of each other (LD r^2^ < 0.001 within 10,000 kb). In MVMR analyses, the IVs were the combination of SNPs that were at a genome-wide significant level (*p* < 5.00 × 10^−8^) in either the GWAS of relative carbohydrate intake or the GWAS of each mediator and were independent of each other (LD r^2^ < 0.001 within 10,000 kb).

#### 2.2.3. Outcome

Summary-level GWAS data for hypertension were from two European cohorts: the FinnGen Study and the UK Biobank. The FinnGen Study is a Finnish, nationwide GWAS meta-analysis of 13 cohorts and biobanks, which has no overlap with the GWASs for relative carbohydrate intake or mediators to guarantee the lowest type 1 error rate [22]. The FinnGen Study included 42,857 participants with hypertension, defined as the presence of essential hypertension, and 162,837 participants without essential hypertension or any other hypertensive diseases as controls [22]. The International Classification of Diseases diagnosis codes used to define essential hypertension are provided in Supplementary Table S3 [22]. The UK Biobank is a cohort study that included over 500,000 men and women from the UK general population between 2006 and 2010 [23]. GWAS data for hypertension from the UK Biobank were based on 77,723 cases of essential hypertension (PheCode 401.1) and 330,366 controls without essential hypertension or any other hypertensive diseases. To ensure the credibility of the results, we extracted the genetic associations of IVs with hypertension from the FinnGen Study for discovery and applied the large sample size of the UK Biobank for supplementary material and to maximize statistical power.

### 2.3. Statistical Analysis

#### 2.3.1. UVMR and MVMR Analyses

We applied UVMR to assess the total causal effects of relative carbohydrate intake, protein intake, and fat intake on hypertension as well as the causal effect of hypertension on relative carbohydrate intake. We performed MVMR to assess the independent causal effect of relative carbohydrate intake on hypertension with adjustment for other macronutrient intakes that had a causal effect on hypertension. All MR analyses fulfilled three core assumptions: (1) the genetic variants must be strongly associated with the exposure in UVMR and at least one of the multiple exposures in MVMR; (2) the genetic variants must not be associated with confounders of the associations between exposures and outcomes; and (3) the effects of genetic variants on outcomes must go through exposures [24]. Where IVs for the exposure (e.g., relative carbohydrate intake) were not available in summary-level statistics of the outcome (e.g., hypertension), we replaced them with proxy SNPs in high LD (r^2^ > 0.8) identified on the online platform LDlink (https://ldlink.nci.nih.gov/; accessed on 28 May 2023) [25]. We used the random-effect inverse-variance weighted (IVW) method as the main analysis in UVMR and the multivariable IVW (MV-IVW) as the main analysis in MVMR. 

#### 2.3.2. Mediation MR Analyses

We performed a two-step MR to evaluate potential mediators of the association between relative carbohydrate intake and hypertension [26]. The first step was to estimate the causal effect of relative carbohydrate intake on each psychological well-being indicator and adiposity trait using UVMR (β1). Reverse MR of each mediator with hypertension was conducted to determine if there was bidirectionality that might affect the validity of the mediation model. The second step was to assess the causal effect of each mediator on hypertension using MVMR with adjustment for relative carbohydrate intake (β2), which was on the premise that each mediator was causally associated with hypertension in UVMR. The proportion mediated by each mediator in the causal association between relative carbohydrate intake and hypertension was calculated as the product of β1 and β2 divided by the total causal effect. The 95% confidence intervals (CIs) of the mediation proportions were calculated using the delta method [27].

#### 2.3.3. MR Sensitivity Analyses

In UVMR, we applied the weighted median, weighted mode, MR-Egger, and MR pleiotropy residual sum and outlier (MR-PRESSO) methods as sensitivity analyses based on different assumptions. The weighted median method provides reliable causal estimates allowing for up to 50% of genetic variants to violate the MR assumption under the presence of horizontal pleiotropy [28]. The weighted mode method indicates reliable causal results if the SNPs contributing to the largest cluster are valid [29]. In the MR-Egger analyses, the MR-Egger intercept test is used to detect potential horizontal pleiotropy, and the slope coefficient from the MR-Egger regression is a consistent estimate of the causal effect in the presence of horizontal pleiotropy [30]. The MR-PRESSO method detects potential horizontally pleiotropic SNPs and generates causal estimates after exclusion of the identified outlying SNPs [31]. In addition, in reverse MR assessing the effect of hypertension on relative carbohydrate intake, we conducted the MR Steiger test of directionality to ascertain whether the estimate of causal direction was accurate [32]. In MVMR, we performed the MVMR-median, MVMR-Egger, and MVMR-Lasso analyses to validate the robustness of the MV-IVW results [33]. The F statistics were used to evaluate the validity of the IVs, and Cochran’s Q statistics were applied to assess the heterogeneity between the IVs. All the MR analyses were conducted using R packages TwoSampleMR (version 0.5.7), MVMR (version 0.4), and MRPRESSO (version 1.0) in R software (version 4.3.1; R Development Core Team, Vienna, Austria). A *p* value < 0.05 indicates statistical significance. The IVW estimates were considered as causal associations only if they had the same direction and statistical significance as at least one sensitivity analysis with no evidence of pleiotropy (*p* for Egger intercept > 0.05). All results were presented as odds ratios (ORs), β coefficients, or proportions, with corresponding 95% CIs.

#### 2.3.4. Meta-Analyses of Estimates from Two Outcome Databases

All MR analyses for the causal effect of each exposure and mediator on hypertension were performed separately in the databases of FinnGen and UK Biobank. We calculated the I2 statistics and corresponding *p* values derived from Cochran’s Q test to quantify the heterogeneity between estimates from the two databases. Given the absence of heterogeneity, fixed-effect model meta-analyses were used to pool the results for hypertension from the two databases. All meta-analyses were performed using R package meta (version 6.1-0).

## 3. Results

### 3.1. Effect of Relative Carbohydrate Intake on Hypertension and the Reverse Effect

**Figure 2:**
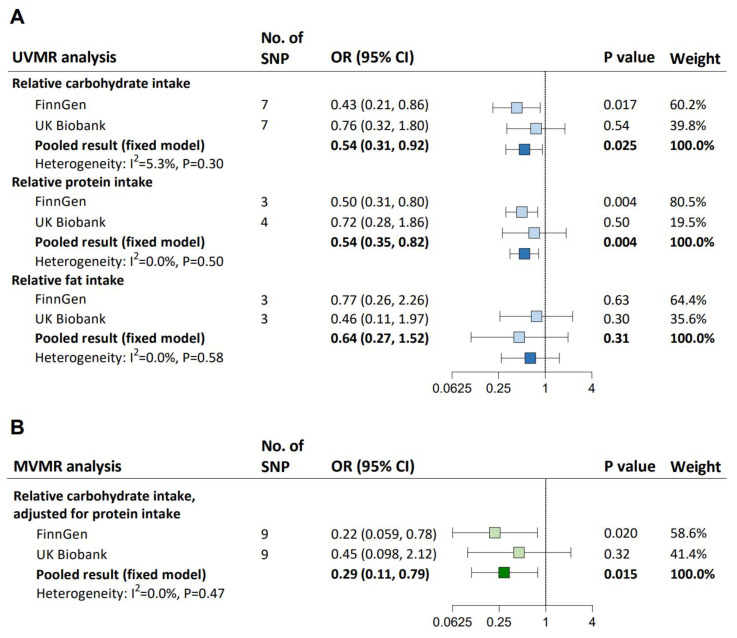
UVMR and MVMR estimates for the causal association between relative carbohydrate intake and hypertension. (**A**) UVMR assessing the causal effects of relative carbohydrate intake, protein intake, and fat intake on hypertension. (**B**) MVMR assessing the independent causal effect of relative carbohydrate intake on hypertension. The blue squares represent UVMR estimates for the effects of macronutrient intakes on hypertension, and the green squares represent MVMR estimates for the effects of macronutrient intakes on hypertension, with light ones representing the results from FinnGen or UK Biobank and dark ones representing the pooled results. ORs (95% CIs) were based on the IVW (UVMR) and MV-IVW (MVMR) analyses, indicating the risk for hypertension per 1-SD increase in relative carbohydrate, fat, or protein intake. CI, confidence interval; IVW, inverse variance weighted; MV-IVW, multivariable inverse variance weighted; MVMR, multivariable Mendelian randomization; OR, odds ratio; SD, standard deviation; SNP, single nucleotide polymorphism; UVMR, univariable Mendelian randomization.

In UVMR, each one-standard deviation (SD) increase in genetically determined carbohydrate intake (pooled IVW-estimated OR: 0.54; 95% CI: 0.31–0.92) and protein intake (0.54; 0.35–0.82), but not fat intake, was causally associated with a lower risk of hypertension (Figure 2A). All UVMR estimates were validated by at least one sensitivity analysis (Supplementary Table S4). We found heterogeneity between IVs but no evidence of weak instrument bias (all F statistics ≥ 50) or horizontal pleiotropy affecting the MR results (all *p* for Egger intercept ≥ 0.21; Supplementary Table S5). The causal association between each one-SD increase in relative carbohydrate intake and hypertension remained after adjusting for relative protein intake (pooled MV-IVW-estimated OR: 0.29; 95% CI: 0.11–0.79; Figure 2B), which was supported by the MVMR-median and MVMR-Lasso results (Supplementary Table S6). There was heterogeneity between IVs, but the instrumental validity test suggested sufficient instrument strength (both F statistics = 33), and no horizontal pleiotropy was detected (both *p* for Egger intercept ≥ 0.48).

We found no evidence of a causal effect of hypertension on carbohydrate intake across all MR methods, and the F statistic suggested no weak instrument bias (Supplementary Table S7). The MR Steiger test of directionality ascertained the unidirectional causality from carbohydrate intake to hypertension (Supplementary Table S8). There was heterogeneity between IVs but no horizontal pleiotropy (Supplementary Table S9).

### 3.2. Effect of Relative Carbohydrate Intake on Psychological Well-Being and Adiposity

Each one-SD increase in genetically determined relative carbohydrate intake was associated with better psychological well-being, including higher levels of positive affect (IVW-estimated β: 0.171; 95% CI: 0.063–0.278) and life satisfaction (0.183; 0.069–0.298), lower levels of neuroticism (−0.171; −0.270, −0.073) and depressive sym ptoms (−0.145; −0.235, −0.056), and lower MDD risk (IVW-estimated OR: 0.60; 95% CI: 0.48–0.75), as well as less adiposity, including lower levels of BMI (β: −0.669 SDs; 95% CI: −1.006, −0.332), WHR (−0.357 SDs; −0.562, −0.152), WC (−0.498 SDs; −0.706, −0.290), HC (−0.468 SDs; −0.739, −0.197), and BF% (−0.427 SDs; −0.771, −0.082); at least two sensitivity analyses confirmed these IVW estimates (Table 2). Reverse MR analyses showed no causal associations of psychological well-being and adiposity with relative carbohydrate intake, except for a causal effect of each 1-SD higher BF% on 0.079 (95% CI: 0.044–0.114) SDs lower relative carbohydrate intake (Supplementary Table S10). There was heterogeneity among IVs but no horizontal pleiotropy (all *p* for Egger intercept ≥ 0.22; Supplementary Table S11).

### 3.3. Effects of Psychological Well-Being and Adiposity on Hypertension

**Figure 3:**
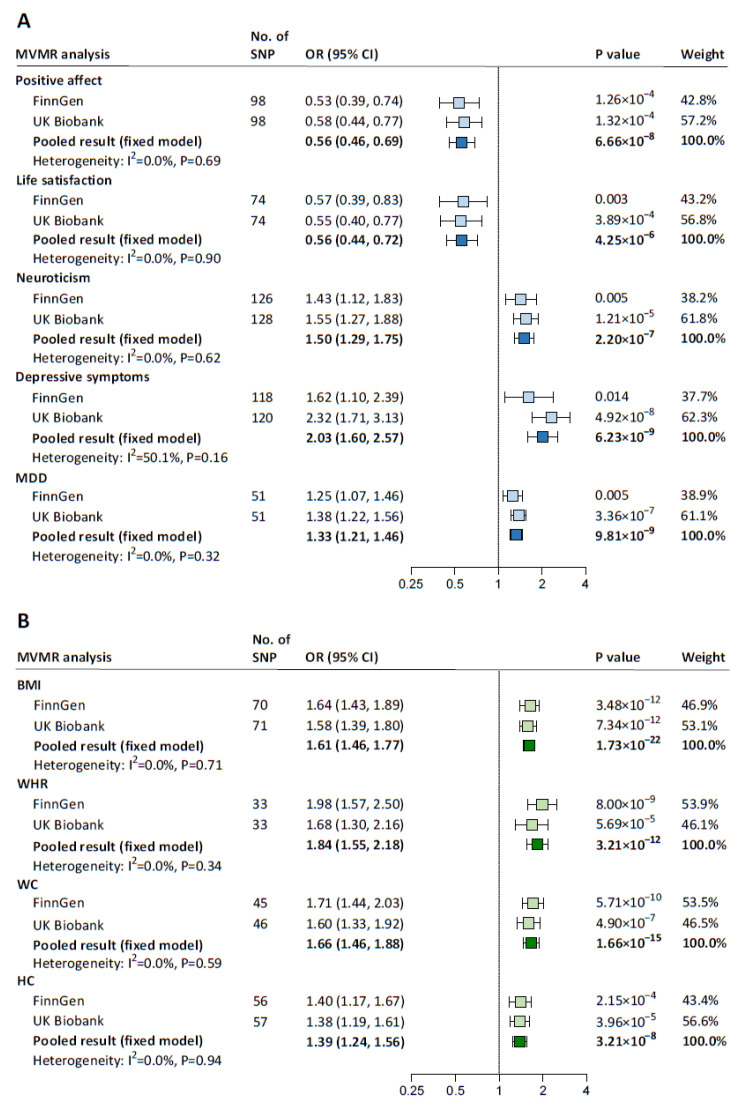
MVMR estimates for the causal association between each mediator and hypertension with adjustment for relative carbohydrate intake. (**A**) MVMR assessing the direct causal effect of each psychological well-being indicator on hypertension with adjustment for relative carbohydrate intake. (**B**) MVMR assessing the direct causal effect of each adiposity trait (BF% was excluded from the mediation analyses for its reverse causal association with relative carbohydrate intake) on hypertension with adjustment for relative carbohydrate intake. The blue squares represent MVMR estimates for the direct causal effect of each psychological well-being indicator on hypertension with adjustment for relative carbohydrate intake, and the green squares represent MVMR estimates for the direct causal effect of each adiposity trait on hypertension with adjustment for relative carbohydrate intake, with light ones representing the results from FinnGen or UK Biobank and dark ones representing the pooled results. ORs (95% CIs) were based on the MV-IVW analyses, indicating the risk for hypertension associated with each psychological well-being indicator or each 1-SD increase in each adiposity trait. BF%, body fat percentage; BMI, body mass index; CI, confidence interval; HC, hip circumference; MDD, major depressive disorder; MV-IVW, multivariable inverse variance weighted; MVMR, multivariable Mendelian randomization; OR, odds ratio; SD, standard deviation; SNP, single nucleotide polymorphism; WC, waist circumference; WHR, waist-to-hip ratio.

BF% was excluded from the mediation analyses due to its reverse causal effect on relative carbohydrate intake (Supplementary Table S10). UVMR results for the associations of the remaining five psychological well-being indicators and four adiposity traits with hypertension are presented in Supplementary Table S12. In MVMR, with adjustment for relative carbohydrate intake, positive affect (pooled IVW-estimated OR: 0.56; 95% CI: 0.46–0.69) and life satisfaction (0.56; 0.44–0.72) were associated with a lower risk of hypertension, whereas neuroticism (1.50; 1.29–1.75), depressive symptoms (2.03; 1.60–2.57), and MDD (1.33; 1.21–1.46) were associated with a higher risk of hypertension (Figure 3A). Each one-SD increase in each adiposity trait was causally associated with a higher risk of hypertension independently of relative carbohydrate intake, with ORs (95% CIs) ranging from 1.84 (1.55–2.18) for WHR to 1.39 (1.24–1.56) for HC (Figure 3B). All MV-IVW estimates were supported by the MVMR-Egger method (Supplementary Table S13). The instrumental validity test indicated no evidence of weak instrument bias (all F statistics ≥ 28); there was heterogeneity among IVs but no horizontal pleiotropy (all *p* for Egger intercept ≥ 0.12).

### 3.4. Mediating Effects of Psychological Well-Being and Adiposity

**Figure 4:**
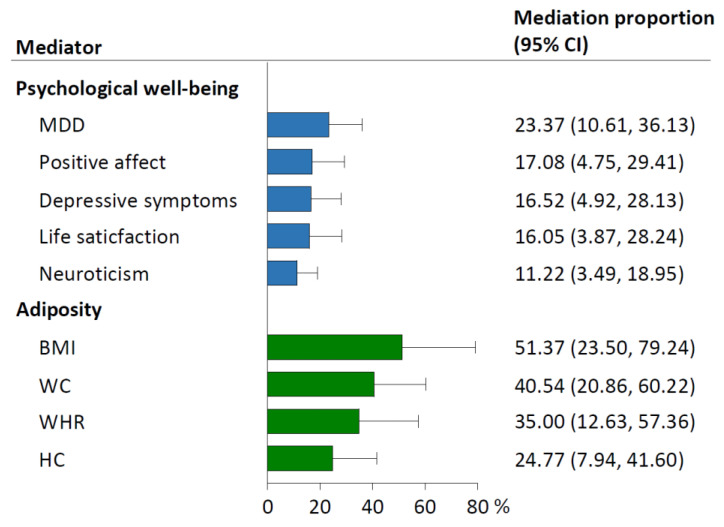
Mediation proportion of each mediator in the causal association between relative carbohydrate intake and hypertension. Mediation proportion (95% CI) for each mediator was obtained from the two-step MR analyses. The effect sizes of relative carbohydrate intake and each mediator on hypertension used to calculate the mediation proportions (95% CIs) were pooled results from FinnGen and UK Biobank by meta-analyses using the fixed-effect model. BMI, body mass index; CI, confidence interval; HC, hip circumference; MDD, major depressive disorder; MR, Mendelian randomization; WC, waist circumference; WHR, waist-to-hip ratio.

Of the five psychological well-being indicators, MDD mediated the largest proportion (23.37%; 95% CI: 10.61–36.13%) of the causal effect of relative carbohydrate intake on hypertension, followed by positive affect (17.08%; 4.75–29.41%), depressive symptoms (16.52%; 4.92–28.13%), life satisfaction (16.05%; 3.87–28.24%), and neuroticism (11.22%; 3.49–18.95%; Figure 4). The four adiposity traits ranked by mediation proportions included BMI (51.37%; 95% CI: 23.50–79.24%), WC (40.54%; 20.86–60.22%), WHR (35.00%; 12.63–57.36%), and HC (24.77%; 7.94–41.60%).

## 4. Discussion

This MR study provided novel evidence that genetically determined that each one-SD increase in relative carbohydrate intake (mean energy from carbohydrates ranging from 42% to 51%) was causally associated with a 71% lower risk of hypertension, independent of the causal effect of other macronutrients, whereas hypertension showed no reverse causal effect on carbohydrate intake. More importantly, the protective effect of higher relative carbohydrate intake on hypertension was causally mediated by better psychological well-being and less adiposity, with individual mediation proportions ranging from 11.22% to 23.37% for five psychological well-being indicators and 24.77% to 51.37% for four adiposity traits. Our findings elaborated on the independent causal protective role of relative carbohydrate intake in hypertension and the considerable mediating effects of psychological well-being and adiposity in the pathogenesis from carbohydrate intake to hypertension. Of note, the clinical significance of the observed causal effects needs to be interpreted in the context of public health, considering the absolute risk reduction alongside the relative risk to determine its practical importance for hypertension prevention and intervention.

Noteworthily, in this study, the beneficial role of carbohydrate intake in hypertension might be dependent on the quantity and quality of carbohydrate intake. In our study, we applied genetic variants as an unconfounded proxy for total energy-adjusted relative carbohydrate intake in European-ancestry individuals from Europe and North America [14]. It has been documented that the percentage of energy from carbohydrate intake was lower in European and North American cohorts (mean values generally ≤50%) than in Asian or Chinese cohorts (mean values generally >60%) [1,14,34]. Therefore, our findings are not inconsistent with the previous China Health and Nutrition Survey, which showed a U-shaped association between carbohydrate intake and hypertension, with an optimal effect at the carbohydrate-to-energy proportion of 50–55% for individuals without hypertension [5]. It is possible that the inverse association between carbohydrate intake and hypertension in our study primarily represented the left side of the U-shaped relationship reported in the Chinese cohort [5]. Moreover, because the consumption of high-quality carbohydrates, such as non-starchy vegetables, whole fruits, legumes, and whole-kernel grains, has been higher in Europe and North America than in other regions [35], our results were in accordance with the previously identified favorable effects of high-quality carbohydrate intake on hypertension and cardiovascular disease [5,7]. On the other hand, our study indicated that the genetic liability to hypertension might not affect carbohydrate intake, confirming the unidirectional causality from carbohydrate intake to hypertension.

Possible biological mechanisms underlying the causal effect of carbohydrate intake on hypertension were mainly the alterations of the hypothalamic–pituitary–adrenal (HPA) axis. Hyperfunction of the HPA axis has been implicated in the pathogenesis of psychological disorders and hypertension [36,37], and its subsequent effects on elevated cortisol concentrations have been associated with fat accumulation and weight gain [37]. Conversely, carbohydrate-rich diets can lead to lower HPA axis stress responses [9], thereby ameliorating HPA axis-related psychological dysfunction and hypertension.

In this context, our study identified two causal mediating pathways from carbohydrate intake to hypertension. Overall, adiposity traits conferred higher mediation proportions than psychological well-being indicators in this study. Our findings were in line with a previous MR study that higher carbohydrate intake was associated with lower levels of BMI and WC [10] and added new evidence to the associations of higher relative carbohydrate intake with lower levels of WHR, HC, and BF%. After excluding BF%, which exhibited a bidirectional association with carbohydrate intake, of the remaining four adiposity mediators, BMI showed the greatest mediation proportion with itself mediating half (51%) of the effect of carbohydrate intake on hypertension. Previous evidence has revealed that BMI was a major causal risk factor for hypertension and a considerable mediator of the effects of other exposures, such as educational attainment, on hypertension [11]. Our findings further suggested that interventions targeting adiposity, particularly BMI, could gain substantial benefits in reducing hypertension risk attributable to lower carbohydrate intake.

Intriguingly, we for the first time ascertained the beneficial causal effect of better psychological well-being on a lower hypertension risk, extending reliable evidence for the protective role of psychological health in noncommunicable diseases [38,39]. In addition, we remarked a higher positive affect (17%) and life satisfaction (16%) and lower neuroticism (11%) and depressive symptoms (17%), as well as less MDD (23%), each of which mediated a substantial proportion of the total causal effect of carbohydrate intake on hypertension. Our findings suggested that preventive strategies for hypertension might be enhanced not only by reducing neuroticism or depression but also by promoting positive psychological well-being, which can be initiated by modification of dietary behaviors such as improving carbohydrate intake.

This study shed light on the etiology of hypertension and outlined causal pathways of psychological well-being and adiposity that mediated the effect of carbohydrate intake on hypertension, which complied with the emerging concept that the body, mind, and heart are interconnected and interdependent in a relationship, namely the mind–heart–body connection [39]. Of note, increasing evidence has recommended a vicious cycle of psychological health and adiposity, between which the biochemically linked mechanisms include overlapping genetic bases, alterations in systems involved in homeostatic adjustments (e.g., HPA axis), and brain circuitries integrating homeostatic and mood regulatory responses [40,41]. Therefore, in this study, the mediation proportion of each psychological well-being indicator or adiposity trait should be interpreted individually in practical scenarios.

To the best of our knowledge, this study for the first time illustrated the independent causal effect of relative carbohydrate intake on hypertension and quantified the mediating roles of psychological well-being and adiposity in the association. This study has two main strengths. First, we applied a rigorous MR study design to enhance the robustness of MR results, including the strict IV selection such as pleiotropic SNP exclusion based on Phenoscanner search, the normative and specific criteria for mediator evaluation, and the robust causal evidence inferred from the main analysis supported by various sensitivity analyses. Second, we extracted genetic associations of IVs with hypertension from two large cohorts, respectively, comprising a total of 613,783 participants with 120,580 hypertension cases, and meta-analyzed the causal estimates from the two databases that had no evidence of heterogeneity. Therefore, the precision and reliability of the final MR results were largely improved. This study also has several limitations. First, although the concordance of the results from multiple sensitivity analyses and complementary tests indicated that weak IV, horizontal pleiotropy, and outliers that might violate the basic MR assumptions did not significantly influence our causal estimates, the causal associations should be interpreted with caution as several assumptions of the methods are untestable, and heterogeneity of the IVs and residual confounding might still potentially bias some results. The MR results should be interpreted alongside the results from observational studies to gain a deeper understanding of the findings. Second, we were unable to test for a nonlinear causal association between carbohydrate intake and hypertension, which required individual-level data [42]. However, because the study population was from Europe and North America, where the quality and quantity of dietary carbohydrate intake was restricted to a relatively uniform range, the inverse association between carbohydrate intake and hypertension in this study was relatively reliable. Third, specific dietary characteristics such as carbohydrate quality (e.g., distinguishing carbohydrates by glycemic load or fiber content) and meal timing were not captured by the genetic instruments in this study, which may add more precise information on the association between diet and health [3]. Fourth, the potential interactions (e.g., exposure–mediator interaction) cannot be modeled in the present two-step, two-sample MR setting. Nevertheless, the MR approach uses genetic variants fixed at conception and naturally randomly assigned to individuals as a proxy for the exposure and the mediator [8], which could largely alleviate the potential bias caused by the interactions. Fifth, this MR study was based on the GWAS data of European-ancestry individuals. Thus, further investigations in other ethnic populations and utilizing alternative study designs are warranted to strengthen the evidence for causality and generalizability of our findings.

## 5. Conclusions

In summary, this MR study elucidated an independent favorable effect of higher relative carbohydrate intake on hypertension, which was substantially mediated by better psychological well-being and less adiposity. Our findings provide novel evidence for causal risk factors and pathological pathways of hypertension and encourage an integrated and precise approach to promote dietary behavior, psychological well-being, and weight control to tackle the rapid epidemic and heavy burden of hypertension.

## Figures and Tables

**Table 1:** Overview of the GWAS data used in the study.

Phenotype	Unit	Sample Size (Case/Control)	Ancestry	Consortium or Cohort Study	Data Source
Exposure					
Relative carbohydrate intake	1-SD	268,922	European	UK Biobank, DietGen, 14 studies	Meddens SFW et al., 2021 (PMID: 32393786) [14]
Covariate					
Relative protein intake	1-SD	268,922	European	UK Biobank, DietGen, 14 studies	Meddens SFW et al., 2021 (PMID: 32393786) [14]
Relative fat intake	1-SD	268,922	European		
Outcome					
Hypertension	Event	42,857/162,837	European	FinnGen	https://FinnGen.gitbook.io/documentation/ (accessed on 14 May 2023)
	Event	77,723/330,366	European	UK Biobank	https://pan.ukbb.broadinstitute.org/ (accessed on 14 May 2023)
Mediator					
Psychological well-being					
Positive affect	Z score	410,603	European	Meta	Baselmans BML et al., 2019 (PMID: 30643256) [15]
Life satisfaction	Z score	80,852	European		
Neuroticism	Z score	582,989	European		
Depressive symptoms	Z score	1,295,946	European		
MDD	Event	170,756/329,443	European	PGC	Howard DM et al., 2019 (PMID: 30718901) [16]
Adiposity					
BMI	1-SD (4.77 kg/m^2^)	322,154	European	GIANT	Locke AE et al., 2015 (PMID: 25673413) [17]
WHR	1-SD (0.076)	212,244	European		Shungin D et al., 2015 (PMID: 25673412) [18]
WC	1-SD (12.52 cm)	232,101	European		
HC	1-SD (8.45 cm)	213,038	European		
BF%	1-SD	454,633	European	UK Biobank	https://gwas.mrcieu.ac.uk/datasets/ (accessed on 14 May 2023)

BF%, body fat percentage; BMI, body mass index; GIANT, Genetic Investigation of ANthropometric Traits; GWAS, genome-wide association study; HC, hip circumference; MDD, major depressive disorder; PGC, Psychiatric Genomic Consortium; SD, standard deviation; WC, waist circumference; WHR, waist-to-hip ratio.

**Table 2:** UVMR estimates of the causal association between relative carbohydrate intake and each mediator.

Mediator	Method	No. of SNP	β (95% CI) ^1^	OR (95% CI) ^1^	*p* Value
Psychological well-being					
Positive affect	IVW	5	0.171 (0.063, 0.278)	/	0.002
	Weighted median		0.143 (0.037, 0.250)	/	0.008
	Weighted mode		0.140 (0.021, 0.259)	/	0.082
	MR-Egger		0.225 (−0.585, 1.035)	/	0.62
	MR-PRESSO (no outliers)		0.171 (0.063, 0.278)	/	0.036
Life satisfaction	IVW	5	0.183 (0.069, 0.298)	/	0.002
	Weighted median		0.157 (0.039, 0.276)	/	0.009
	Weighted mode		0.155 (0.025, 0.285)	/	0.079
	MR-Egger		0.258 (−0.584, 1.110)	/	0.59
	MR-PRESSO (no outliers)		0.183 (0.069, 0.298)	/	0.035
Neuroticism	IVW	5	−0.171 (−0.270, −0.073)	/	6.69 × 10^−4^
	Weighted median		−0.179 (−0.299, −0.059)	/	0.004
	Weighted mode		−0.172 (−0.312, −0.032)	/	0.073
	MR-Egger		−0.691 (−1.368, −0.014)	/	0.14
	MR-PRESSO (no outliers)		−0.171 (−0.270, −0.073)	/	0.027
Depressive symptoms	IVW	5	−0.145 (−0.235, −0.056)	/	0.001
	Weighted median		−0.110 (−0.184, −0.035)	/	0.004
	Weighted mode		−0.111 (−0.189, −0.034)	/	0.048
	MR-Egger		−0.126 (−0.855, 0.603)	/	0.76
	MR-PRESSO (no outliers)		−0.145 (−0.235, −0.056)	/	0.034
MDD	IVW	7	−0.512 (−0.731, −0.294)	0.60 (0.48, 0.75)	4.15 × 10^−6^
	Weighted median		−0.541 (−0.802, −0.281)	0.58 (0.45, 0.76)	4.71 × 10^−5^
	Weighted mode		−0.528 (−0.948, −0.107)	0.59 (0.39, 0.90)	0.049
	MR-Egger		0.255 (−0.845, 1.355)	1.29 (0.43, 3.88)	0.67
	MR-PRESSO (no outliers)		−0.512 (−0.731, −0.294)	0.60 (0.48, 0.75)	0.004
Adiposity					
BMI	IVW	5	−0.669 (−1.006, −0.332)	/	1.01 × 10^−4^
	Weighted median		−0.665 (−0.960, −0.369)	/	1.08 × 10^−5^
	Weighted mode		−0.859 (−1.454, −0.264)	/	0.047
	MR-Egger		−1.461 (−4.187, 1.266)	/	0.37
	MR-PRESSO (no outliers)		−0.669 (−1.006, −0.332)	/	0.018
WHR	IVW	5	−0.357 (−0.562, −0.152)	/	6.35 × 10^−4^
	Weighted median		−0.345 (−0.596, −0.094)	/	0.007
	Weighted mode		−0.320 (−0.642, 0.001)	/	0.12
	MR-Egger		−0.613 (−2.146, 0.920)	/	0.49
	MR-PRESSO (no outliers)		−0.357 (−0.415, −0.299)	/	2.67 × 10^−4^
WC	IVW	5	−0.498 (−0.706, −0.290)	/	2.80 × 10^−6^
	Weighted median		−0.447 (−0.734, −0.160)	/	0.002
	Weighted mode		−0.331 (−0.768, 0.107)	/	0.21
	MR-Egger		−0.905 (−2.539, 0.729)	/	0.36
	MR-PRESSO (no outliers)		−0.498 (−0.694, −0.303)	/	0.008
HC	IVW	5	−0.468 (−0.739, −0.197)	/	7.22 × 10^−4^
	Weighted median		−0.354 (−0.697, −0.010)	/	0.044
	Weighted mode		−0.275 (−0.869, 0.318)	/	0.41
	MR-Egger		−0.617 (−2.925, 1.692)	/	0.64
	MR-PRESSO (no outliers)		−0.468 (−0.739, −0.197)	/	0.028
BF%	IVW	7	−0.427 (−0.771, −0.082)	/	0.015
	Weighted median		−0.406 (−0.542, −0.270)	/	4.56 × 10^−9^
	Weighted mode		−0.388 (−0.532, −0.244)	/	0.002
	MR-Egger		−0.939 (−2.919, 1.043)	/	0.40
	MR-PRESSO (3 outliers)		−0.408 (−0.500, −0.315)	/	0.003

^1^ Causal estimates of odds for MDD or changes in z score of the rest four psychological well-being indicators or changes in SDs of adiposity traits associated with each 1-SD increase in relative carbohydrate intake. BF%, body fat percentage; BMI, body mass index; CI, confidence interval; HC, hip circumference; IVW, inverse-variance weighted; MDD, major depressive disorder; MR, Mendelian randomization; OR, odds ratio; PRESSO, pleiotropy residual sum and outlier; SD, standard deviation; SNP, single nucleotide polymorphism; UVMR, univariable Mendelian randomization; WC, waist circumference; WHR, waist-to-hip ratio.

## Data Availability

All the summary-level GWAS data used in the analyses are publicly available as shown in Table 1. All data generated in the current study could be obtained from the Supplementary Materials.

## Supplementary Materials

The following supporting information can be downloaded at https://www.mdpi.com/article/10.3390/nu15224817/s1. Table S1: Potential confounders of dietary macronutrient intake-associated SNPs under the condition of *p* < 5 × 10^−8^ in the PhenoScanner database. Table S2: Summary information of SNPs used as genetic instruments for dietary macronutrient intake in this MR study. Table S3: Overview of the used ICD diagnosis codes to define cases with essential hypertension and controls in the Fifth release of the FinnGen Study. Table S4: UVMR estimates for the causal associations of relative carbohydrate intake, protein intake, and fat intake with hypertension. Table S5: Directional pleiotropy test and heterogeneity test for the causal associations of relative carbohydrate intake, protein intake, and fat intake with hypertension. Table S6: MVMR estimates for the causal association between relative carbohydrate intake and hypertension with adjustment for relative protein intake. Table S7: UVMR estimates for the causal association between hypertension and relative carbohydrate intake. Table S8: MR Steiger test of directionality in the MR analysis for the effect of hypertension on relative carbohydrate intake. Table S9: Directional pleiotropy test and heterogeneity test for the causal association between hypertension and relative carbohydrate intake. Table S10: UVMR estimates for the causal association between each mediator and relative carbohydrate intake. Table S11: Directional pleiotropy test and heterogeneity test for the causal association between relative carbohydrate intake and each mediator. Table S12: UVMR estimates for the causal association between each mediator and hypertension. Table S13: MVMR estimates for the causal association between each mediator and hypertension with adjustment for relative carbohydrate intake. Figure S1: Flow chart of the present MR study.

## References

[1] Dehghan M., Mente A., Zhang X., Swaminathan S., Li W., Mohan V., Iqbal R., Kumar R., Wentzel-Viljoen E., Rosengren A. (2017). Associations of fats and carbohydrate intake with cardiovascular disease and mortality in 18 countries from five continents (PURE): A prospective cohort study. Lancet.

[2] Hou W., Han T., Sun X., Chen Y., Xu J., Wang Y., Yang X., Jiang W., Sun C. (2022). Relationship Between Carbohydrate Intake (Quantity, Quality, and Time Eaten) and Mortality (Total, Cardiovascular, and Diabetes): Assessment of 2003–2014 National Health and Nutrition Examination Survey Participants. Diabetes Care.

[3] Zhou B., Perel P., Mensah G.A., Ezzati M. (2021). Global epidemiology, health burden and effective interventions for elevated blood pressure and hypertension. Nat. Rev. Cardiol..

[4] NCD Risk Factor Collaboration (NCD-RisC) (2021). Worldwide trends in hypertension prevalence and progress in treatment and control from 1990 to 2019: A pooled analysis of 1201 population-representative studies with 104 million participants. Lancet.

[5] Li Q., Liu C., Zhang S., Li R., Zhang Y., He P., Zhang Z., Liu M., Zhou C., Ye Z. (2021). Dietary Carbohydrate Intake and New-Onset Hypertension: A Nationwide Cohort Study in China. Hypertension.

[6] Ge L., Sadeghirad B., Ball G.D.C., da Costa B.R., Hitchcock C.L., Svendrovski A., Kiflen R., Quadri K., Kwon H.Y., Karamouzian M. (2020). Comparison of dietary macronutrient patterns of 14 popular named dietary programmes for weight and cardiovascular risk factor reduction in adults: Systematic review and network meta-analysis of randomised trials. BMJ.

[7] Kelly R.K., Tong T.Y.N., Watling C.Z., Reynolds A., Piernas C., Schmidt J.A., Papier K., Carter J.L., Key T.J., Perez-Cornago A. (2023). Associations between types and sources of dietary carbohydrates and cardiovascular disease risk: A prospective cohort study of UK Biobank participants. BMC Med..

[8] Davies N.M., Holmes M.V., Davey Smith G. (2018). Reading Mendelian randomisation studies: A guide, glossary, and checklist for clinicians. BMJ.

[9] Yao S., Zhang M., Dong S.S., Wang J.H., Zhang K., Guo J., Guo Y., Yang T.L. (2022). Bidirectional two-sample Mendelian randomization analysis identifies causal associations between relative carbohydrate intake and depression. Nat. Hum. Behav..

[10] Freuer D., Meisinger C., Linseisen J. (2021). Causal relationship between dietary macronutrient composition and anthropometric measures: A bidirectional two-sample Mendelian randomization analysis. Clin. Nutr..

[11] Wang Y., Ye C., Kong L., Zheng J., Xu M., Xu Y., Li M., Zhao Z., Lu J., Chen Y. (2023). Independent Associations of Education, Intelligence, and Cognition with Hypertension and the Mediating Effects of Cardiometabolic Risk Factors: A Mendelian Randomization Study. Hypertension.

[12] Kubzansky L.D., Huffman J.C., Boehm J.K., Hernandez R., Kim E.S., Koga H.K., Feig E.H., Lloyd-Jones D.M., Seligman M.E.P., Labarthe D.R. (2018). Positive Psychological Well-Being and Cardiovascular Disease: JACC Health Promotion Series. J. Am. Coll. Cardiol..

[13] Skrivankova V.W., Richmond R.C., Woolf B.A.R., Yarmolinsky J., Davies N.M., Swanson S.A., VanderWeele T.J., Higgins J.P.T., Timpson N.J., Dimou N. (2021). Strengthening the Reporting of Observational Studies in Epidemiology Using Mendelian Randomization: The STROBE-MR Statement. JAMA.

[14] Meddens S.F.W., de Vlaming R., Bowers P., Burik C.A.P., Linnér R.K., Lee C., Okbay A., Turley P., Rietveld C.A., Fontana M.A. (2021). Genomic analysis of diet composition finds novel loci and associations with health and lifestyle. Mol. Psychiatry.

[15] Baselmans B.M.L., Jansen R., Ip H.F., van Dongen J., Abdellaoui A., van de Weijer M.P., Bao Y., Smart M., Kumari M., Willemsen G. (2019). Multivariate genome-wide analyses of the well-being spectrum. Nat. Genet..

[16] Howard D.M., Adams M.J., Clarke T.K., Hafferty J.D., Gibson J., Shirali M., Coleman J.R.I., Hagenaars S.P., Ward J., Wigmore E.M. (2019). Genome-wide meta-analysis of depression identifies 102 independent variants and highlights the importance of the prefrontal brain regions. Nat. Neurosci..

[17] Locke A.E., Kahali B., Berndt S.I., Justice A.E., Pers T.H., Day F.R., Powell C., Vedantam S., Buchkovich M.L., Yang J. (2015). Genetic studies of body mass index yield new insights for obesity biology. Nature.

[18] Shungin D., Winkler T.W., Croteau-Chonka D.C., Ferreira T., Locke A.E., Mägi R., Strawbridge R.J., Pers T.H., Fischer K., Justice A.E. (2015). New genetic loci link adipose and insulin biology to body fat distribution. Nature.

[19] Staley J.R., Blackshaw J., Kamat M.A., Ellis S., Surendran P., Sun B.B., Paul D.S., Freitag D., Burgess S., Danesh J. (2016). PhenoScanner: A database of human genotype-phenotype associations. Bioinformatics.

[20] Kamat M.A., Blackshaw J.A., Young R., Surendran P., Burgess S., Danesh J., Butterworth A.S., Staley J.R. (2019). PhenoScanner V2: An expanded tool for searching human genotype-phenotype associations. Bioinformatics.

[21] Mitchell R.E., Elsworth B.L., Mitchell R.E., Raistrick C.A., Paternoster L., Hemani G., Gaunt T.R. (2019). MRC IEU UK Biobank GWAS Pipeline Version 2.

[22] Kurki M.I., Karjalainen J., Palta P., Sipilä T.P., Kristiansson K., Donner K.M., Reeve M.P., Laivuori H., Aavikko M., Kaunisto M.A. (2023). FinnGen provides genetic insights from a well-phenotyped isolated population. Nature.

[23] Sudlow C., Gallacher J., Allen N., Beral V., Burton P., Danesh J., Downey P., Elliott P., Green J., Landray M. (2015). UK biobank: An open access resource for identifying the causes of a wide range of complex diseases of middle and old age. PLoS Med..

[24] Emdin C.A., Khera A.V., Kathiresan S. (2017). Mendelian Randomization. JAMA.

[25] Machiela M.J., Chanock S.J. (2015). LDlink: A web-based application for exploring population-specific haplotype structure and linking correlated alleles of possible functional variants. Bioinformatics.

[26] Carter A.R., Sanderson E., Hammerton G., Richmond R.C., Davey Smith G., Heron J., Taylor A.E., Davies N.M., Howe L.D. (2021). Mendelian randomisation for mediation analysis: Current methods and challenges for implementation. Eur. J. Epidemiol..

[27] MacKinnon D.P., Lockwood C.M., Hoffman J.M., West S.G., Sheets V. (2002). A comparison of methods to test mediation and other intervening variable effects. Psychol. Methods.

[28] Bowden J., Davey Smith G., Haycock P.C., Burgess S. (2016). Consistent Estimation in Mendelian Randomization with Some Invalid Instruments Using a Weighted Median Estimator. Genet. Epidemiol..

[29] Hartwig F.P., Davey Smith G., Bowden J. (2017). Robust inference in summary data Mendelian randomization via the zero modal pleiotropy assumption. Int. J. Epidemiol..

[30] Bowden J., Davey Smith G., Burgess S. (2015). Mendelian randomization with invalid instruments: Effect estimation and bias detection through Egger regression. Int. J. Epidemiol..

[31] Verbanck M., Chen C.Y., Neale B., Do R. (2018). Detection of widespread horizontal pleiotropy in causal relationships inferred from Mendelian randomization between complex traits and diseases. Nat. Genet..

[32] Hemani G., Tilling K., Davey Smith G. (2017). Orienting the causal relationship between imprecisely measured traits using GWAS summary data. PLoS Genet..

[33] Burgess S., Thompson S.G. (2015). Multivariable Mendelian randomization: The use of pleiotropic genetic variants to estimate causal effects. Am. J. Epidemiol..

[34] Seidelmann S.B., Claggett B., Cheng S., Henglin M., Shah A., Steffen L.M., Folsom A.R., Rimm E.B., Willett W.C., Solomon S.D. (2018). Dietary carbohydrate intake and mortality: A prospective cohort study and meta-analysis. Lancet Public Health.

[35] Miller V., Mente A., Dehghan M., Rangarajan S., Zhang X., Swaminathan S., Dagenais G., Gupta R., Mohan V., Lear S. (2017). Fruit, vegetable, and legume intake, and cardiovascular disease and deaths in 18 countries (PURE): A prospective cohort study. Lancet.

[36] Keller J., Gomez R., Williams G., Lembke A., Lazzeroni L., Murphy G.M., Schatzberg A.F. (2017). HPA axis in major depression: Cortisol, clinical symptomatology and genetic variation predict cognition. Mol. Psychiatry.

[37] Ortiz R., Kluwe B., Lazarus S., Teruel M.N., Joseph J.J. (2022). Cortisol and cardiometabolic disease: A target for advancing health equity. Trends Endocrinol. Metab..

[38] Yusuf S., Joseph P., Rangarajan S., Islam S., Mente A., Hystad P., Brauer M., Kutty V.R., Gupta R., Wielgosz A. (2020). Association of Symptoms of Depression with Cardiovascular Disease and Mortality in Low-, Middle-, and High-Income Countries. JAMA Psychiatry.

[39] Levine G.N., Cohen B.E., Commodore-Mensah Y., Fleury J., Huffman J.C., Khalid U., Labarthe D.R., Lavretsky H., Michos E.D., Spatz E.S. (2021). Psychological Health, Well-Being, and the Mind-Heart-Body Connection: A Scientific Statement from the American Heart Association. Circulation.

[40] Plackett B. (2022). The vicious cycle of depression and obesity. Nature.

[41] Milaneschi Y., Simmons W.K., van Rossum E.F.C., Penninx B.W. (2019). Depression and obesity: Evidence of shared biological mechanisms. Mol. Psychiatry.

[42] Burgess S., Davies N.M., Thompson S.G., EPIC-InterAct Consortium (2014). Instrumental variable analysis with a nonlinear exposure-outcome relationship. Epidemiology.

