# Supplementary material for: Causal Effect of Relative Carbohydrate Intake on Hypertension through Psychological Well-Being and Adiposity: A Mendelian Randomization Study
Source: Nutrients. 2023 Nov 17;15(22):4817. doi: 10.3390/nu15224817 (PMC10674610; doi:10.3390/nu15224817)

Supplementary Material

Causal Effect of Relative Carbohydrate Intake on Hypertension through Psychological Well-Being and Adiposity: A Mendelian Randomization Study

Chaojie Ye,[1,2] Lijie Kong,[1,2] Yiying Wang,[1,2] Chun Dou,[1,2] Min Xu,[1,2] Jie Zheng,[1,2] Ruizhi Zheng,[1,2] Yu Xu,[1,2] Mian Li,[1,2] Zhiyun Zhao,[1,2] Jieli Lu,[1,2] Yuhong Chen,[1,2] Weiqing Wang,[1,2] Yufang Bi,[1,2] Tiange Wang,[1,2,*] Guang Ning[1,2]

Author Affiliations:
[1]Department of Endocrine and Metabolic Diseases, Shanghai Institute of Endocrine and Metabolic Dis-eases, Ruijin Hospital, Shanghai Jiao Tong University School of Medicine, Shanghai 200025, China;
[2]Shanghai National Clinical Research Center for Metabolic Diseases, Key Laboratory for Endocrine and Metabolic Diseases of the National Health Commission of the PR China, Shanghai Key Laboratory for Endocrine Tumor, Ruijin Hospital, Shanghai Jiao Tong University School of Medicine, Shanghai 200025, China

Table of contents

**Supplementary Table S1.** Potential confounders of dietary macronutrient intake-associated SNPs under the condition of P<5e-08 in the PhenoScanner database	………….3
**Supplementary Table S2.** Summary information of SNPs used as genetic instruments for dietary macronutrient intake in this MR study	………….4
**Supplementary Table S3.** Overview of the used ICD diagnosis codes to define cases with essential hypertension and controls in the Fifth release of the FinnGen Study	………….....5
**Supplementary Table S4.** UVMR estimates for the causal associations of relative carbohydrate intake, protein intake, and fat intake with hypertension	………….....6
**Supplementary Table S5.** Directional pleiotropy test and heterogeneity test for the causal associations of relative carbohydrate intake, protein intake, and fat intake with hypertension	………….....7
**Supplementary Table S6.** MVMR estimates for the causal association between relative carbohydrate intake and hypertension with adjustment for relative protein intake	………….....8
**Supplementary Table S7.** UVMR estimates for the causal association between hypertension and relative carbohydrate intake	………….....9
**Supplementary Table S8.** MR Steiger test of directionality in the MR analysis for the effect of hypertension on relative carbohydrate intake	………....10
**Supplementary Table S9.** Directional pleiotropy test and heterogeneity test for the causal association between hypertension and relative carbohydrate intake	………....11
**Supplementary Table S10.** UVMR estimates for the causal association between each mediator and relative carbohydrate intake	………....12
**Supplementary Table S11.** Directional pleiotropy test and heterogeneity test for the causal association between relative carbohydrate intake and each mediator	………....14
**Supplementary Table S12.** UVMR estimates for the causal association between each mediator and hypertension	………....15
**Supplementary Table S13.** MVMR estimates for the causal association between each mediator and hypertension with adjustment for relative carbohydrate intake	………....18
**Supplementary Figure S1.** Flow chart of the present MR study	………....20

Supplementary Table S1. Potential confounders of dietary macronutrient intake-associated SNPs under the condition of P<5e-08 in the PhenoScanner database

Exposure	Excluded SNP	Trait
Relative carbohydrate intake	rs10433500	Alcohol intake frequency, Ever smoked, Past tobacco smoking, Smoking status: previous, Types of physical activity in last 4 weeks: other exercises
	rs1104608	Alcohol intake frequency
	rs36123991	Alcohol intake frequency, Qualifications: college or university degree
	rs7190396	Alcohol intake frequency, Average weekly beer plus cider intake, Average weekly red wine intake, Usual walking pace
	rs838144	Alcohol intake frequency
Relative protein intake	rs13146907	Alcohol intake frequency
	rs55872725	Alcohol intake frequency, Average weekly beer plus cider intake, Usual walking pace
	rs780094	Alcohol intake frequency, Alcohol intake versus 10 years previously
Relative fat intake	rs1229984	Alcohol consumption, Alcohol consumption drinkers vs non-drinkers, Alcohol consumption drinks per week, Alcohol drinker status: never, Alcohol intake frequency, Alcohol intake versus 10 years previously, Average weekly beer plus cider intake, Average weekly red wine intake, Drinking intensity in ever drinkers, Former alcohol drinker

Abbreviation: SNP, single nucleotide polymorphism.

Supplementary Table S2. Summary information of SNPs used as genetic instruments for dietary macronutrient intake in this MR study

SNP	EA	EAF	β	SE	P value
**SNPs for relative carbohydrate intake**					
rs10206338	A	0.5724	-0.01577	0.00326	1.52e-08
rs10510554	T	0.4362	0.01942	0.00289	2.94e-12
rs10962121	T	0.4791	-0.01519	0.00369	3.40e-08
rs2472297	T	0.2055	-0.01795	0.00388	3.73e-08
rs429358	T	0.8592	-0.02698	0.00279	3.49e-12
rs7012637	A	0.4915	0.01734	0.00278	4.68e-10
rs8097672	A	0.847	0.02346	0.00278	1.95e-09
rs10433500[a]	A	0.6301	0.01608	0.00281	1.96e-08
rs1104608[a]	C	0.4184	0.01847	0.00391	1.74e-10
rs36123991[a]	T	0.1858	0.0213	0.00275	8.24e-09
rs7190396[a]	T	0.5985	0.01781	0.00286	2.39e-10
rs838144[a]	T	0.5342	-0.02323	0.0048	3.26e-17
rs9987289[†]	A	0.0904	-0.02623	0.00275	4.64e-08
**SNPs for relative protein intake**					
rs838133	A	0.425	-0.03182	0.00301	4.52e-26
rs445551	A	0.3168	0.01919	0.00339	1.49e-08
rs1603978	A	0.6939	0.01917	0.00299	1.35e-10
rs1461729	A	0.0978	0.03183	0.00459	4.09e-12
rs55872725[a]	T	0.4002	0.01779	0.0028	2.09e-10
rs780094[a]	T	0.3976	0.01752	0.00282	5.58e-10
rs13146907[a]	A	0.6252	-0.0219	0.00284	1.24e-14
**SNPs for relative fat intake**					
rs33988101	T	0.4993	-0.0294	0.00276	1.66e-26
rs57193069	A	0.5526	-0.01574	0.0028	1.80e-08
rs7012814	A	0.4921	-0.0189	0.00278	1.12e-11
rs1229984[a]	T	0.0475	0.09777	0.00886	2.64e-28
rs9927317[†]	C	0.606	-0.0242	0.0035	4.77e-12
rs429358[†]	T	0.8592	0.02379	0.00388	8.65e-10

[a] Excluded for the associations with potential confounders as shown in Supplementary Table 2.

[†] Excluded after LD clumping (LD r^2<0.001 within 10,000 kb).

Abbreviations: EA, effect allele; EAF, effect allele frequency; LD, linkage disequilibrium; MR, Mendelian randomization; SE, standard error; SNP, single nucleotide polymorphism.

Supplementary Table S3. Overview of the used ICD diagnosis codes to define cases with essential hypertension and controls in the Fifth release of the FinnGen Study

**Diagnostic classification code**	**Case**<br>(Participants **with** at least one of the following diagnoses according to the electronic health registries)	**Control**<br>(Participants **without** any of the following diagnoses according to the electronic health registries)
ICD-10	I10	I10, I11, I12, I13, I15, I674
ICD-9	4039A, 4019X	4019X, 4029A, 4029B, 4039A, 4040A, 4059A, 4059B, 4372A, 4059X
ICD-8	401, 402, 403, 404	400, 401, 402, 403, 404

Abbreviation: ICD, International Classification of Diseases.

Supplementary Table S4. UVMR estimates for the causal associations of relative carbohydrate intake, protein intake, and fat intake with hypertension

UVMR analysis	Method	No. of SNP	F statistic	OR (95% CI)[a]	P value
**Relative carbohydrate intake**					
FinnGen	IVW	7	50	0.43 (0.21, 0.86)	0.017
	Weighted median			0.36 (0.19, 0.68)	0.002
	Weighted mode			0.30 (0.14, 0.64)	0.022
	MR-Egger			0.10 (0.002, 4.47)	0.29
	MR-PRESSO (1 outlier)			0.33 (0.21, 0.50)	0.004
UK Biobank	IVW	7	50	0.76 (0.32, 1.80)	0.54
	Weighted median			0.84 (0.53, 1.33)	0.45
	Weighted mode			0.82 (0.43, 1.55)	0.56
	MR-Egger			0.42 (0.003, 63.44)	0.75
	MR-PRESSO (3 outliers)			0.62 (0.37, 1.05)	0.17
**Relative protein intake**					
FinnGen	IVW	3	64	0.50 (0.32, 0.80)	0.004
	Weighted median			0.52 (0.30, 0.87)	0.014
	Weighted mode			0.51 (0.28, 0.94)	0.16
	MR-Egger			1.33 (0.16, 11.27)	0.84
	MR-PRESSO[†]	NA	NA	NA	NA
UK Biobank	IVW	4	58	0.72 (0.28, 1.86)	0.50
	Weighted median			0.62 (0.40, 0.98)	0.042
	Weighted mode			0.45 (0.31, 0.65)	0.024
	MR-Egger			0.69 (0.006, 76.41)	0.89
	MR-PRESSO (2 outliers)			0.73 (0.67, 0.81)	0.099
**Relative fat intake**					
FinnGen	IVW	3	64	0.77 (0.26, 2.26)	0.63
	Weighted median			0.74 (0.40, 1.38)	0.34
	Weighted mode			0.49 (0.25, 0.98)	0.18
	MR-Egger			0.048 (0.007, 0.33)	0.20
	MR-PRESSO[†]	NA	NA	NA	NA
UK Biobank	IVW	3	64	0.46 (0.11, 1.97)	0.30
	Weighted median			0.29 (0.20, 0.44)	2.85e-09
	Weighted mode			0.29 (0.19, 0.43)	0.028
	MR-Egger			0.023 (0.000, 3.10)	0.37
	MR-PRESSO[†]	NA	NA	NA	NA

[a] Causal estimates of odds for hypertension per 1-SD increase in relative carbohydrate, protein, or fat intake.

[†] Not available for not enough instrumental variables.

Abbreviations: CI, confidence interval; IVW, inverse-variance weighted; OR, odds ratio; PRESSO, pleiotropy residual sum and outlier; SD, standard deviation; SNP, single nucleotide polymorphism; UVMR, univariable Mendelian randomization.

Supplementary Table S5. Directional pleiotropy test and heterogeneity test for the causal associations of relative carbohydrate intake, protein intake, and fat intake with hypertension

**UVMR analysis**	**Directional pleiotropy test**			**Heterogeneity test**		
	**Egger intercept**	**SE**	**P value**	**Q statistic**	**Q df**	**P value**
**Relative carbohydrate intake**						
FinnGen	0.028	0.037	0.48	15.92	6	0.014
UK Biobank	0.012	0.048	0.82	57.00	6	1.83e-10
**Relative protein intake**						
FinnGen	-0.028	0.030	0.53	1.36	2	0.51
UK Biobank	0.001	0.061	0.99	32.93	3	3.33e-07
**Relative fat intake**						
FinnGen	0.064	0.022	0.21	8.63	2	0.013
UK Biobank	0.068	0.055	0.43	38.60	2	4.16e-09

Abbreviations: SE, standard error; UVMR, univariable Mendelian randomization.

Supplementary Table S6. MVMR estimates for the causal association between relative carbohydrate intake and hypertension with adjustment for relative protein intake

MVMR analysis	Method	OR (95% CI)[a]	P value	No. of SNP	F statistic	Heterogeneity test		Directional pleiotropy test		
						Q statistic	P value	Egger intercept	SE	P value
**Relative carbohydrate intake, adjusted for protein intake**										
FinnGen	MV-IVW	0.22 (0.059, 0.78)	0.020	9	33	22.74	0.002	0.007	0.026	0.80
	MVMR-median	0.16 (0.046, 0.55)	0.004							
	MVMR-Egger	0.16 (0.012, 2.22)	0.17							
	MVMR-Lasso	0.13 (0.048, 0.38)	1.30e-04							
UK Biobank	MV-IVW	0.45 (0.098, 2.12)	0.32	9	33	75.25	1.27e-13	-0.006	0.031	0.83
	MVMR-median	0.40 (0.14, 1.18)	0.098							
	MVMR-Egger	0.60 (0.026, 13.83)	0.75							
	MVMR-Lasso	0.28 (0.14, 0.58)	5.41e-04							
Combined effect (fixed model)	MV-IVW	0.29 (0.11, 0.79)	0.015	/	/	/	/	/	/	/
	MVMR-median	0.27 (0.12, 0.61)	0.002							
	MVMR-Egger	0.28 (0.037, 2.07)	0.21							
	MVMR-Lasso	0.22 (0.12, 0.40)	4.89e-07							

[a] Causal estimates of odds for hypertension per 1-SD increase in relative carbohydrate intake.
Abbreviations: CI, confidence interval; MV-IVW, multivariable inverse-variance weighted; MVMR, multivariable Mendelian randomization; OR, odds ratio; SD, standard deviation; SE, standard error; SNP, single nucleotide polymorphism.

Supplementary Table S7. UVMR estimates for the causal association between hypertension and relative carbohydrate intake

Method	No. of SNP	F statistic	β (95% CI)[a]	P value
**Hypertension on relative carbohydrate intake**				
IVW	34	49	0.001 (-0.017, 0.020)	0.88
Weighted median			0.004 (-0.016, 0.024)	0.67
Weighted mode			0.009 (-0.028, 0.046)	0.65
MR-Egger			0.030 (-0.040, 0.099)	0.41
MR-PRESSO (1 outlier)			0.007 (-0.006, 0.019)	0.30

[a] Causal estimate of change in SDs of relative carbohydrate intake associated with hypertension.

Abbreviations: CI, confidence interval; IVW, inverse-variance weighted; MR, Mendelian randomization; PRESSO, pleiotropy residual sum and outlier; SD, standard deviation; SNP, single nucleotide polymorphism; UVMR, univariable Mendelian randomization.

Supplementary Table S8. MR Steiger test of directionality in the MR analysis for the effect of hypertension on relative carbohydrate intake

**Exposure**	**Outcome**	**r^2 exposure**	**r^2 outcome**	**Correct causal direction**	**Steiger P value**
Hypertension	Relative carbohydrate intake	0.336	6.51e-04	True	0.00

Abbreviation: MR, Mendelian randomization.

Supplementary Table S9. Directional pleiotropy test and heterogeneity test for the causal association between hypertension and relative carbohydrate intake

		Directional pleiotropy test			Heterogeneity test		
**Exposure**	**Outcome**	**Egger intercept**	**SE**	**P value**	**Q statistic**	**Q df**	**P value**
Hypertension	Relative carbohydrate intake	-0.002	0.003	0.41	53.09	33	0.015

Abbreviation: SE, standard error.

Supplementary Table S10. UVMR estimates for the causal association between each mediator and relative carbohydrate intake

Mediator	Method	No. of SNP	β (95% CI)[a]	P value	Directional pleiotropy test			Heterogeneity test		
					Egger intercept	SE	P value	Q statistic	Q df	P value
**Each mediator on relative carbohydrate intake**										
Positive affect	IVW	93	0.034 (-0.076, 0.143)	0.55	-0.001	0.002	0.61	225.93	92	2.89e-13
	Weighted median		0.030 (-0.083, 0.144)	0.60						
	Weighted mode		-0.017 (-0.247, 0.213)	0.88						
	MR-Egger		0.149 (-0.302, 0.600)	0.52						
	MR-PRESSO (3 outliers)		0.047 (-0.050, 0.143)	0.35						
Life satisfaction	IVW	68	0.056 (-0.063, 0.174)	0.36	0.001	0.003	0.62	148.87	67	3.72e-08
	Weighted median		0.029 (-0.096, 0.154)	0.65						
	Weighted mode		0.057 (-0.251, 0.364)	0.72						
	MR-Egger		-0.089 (-0.673, 0.495)	0.77						
	MR-PRESSO (2 outliers)		0.100 (-0.009, 0.210)	0.077						
Neuroticism	IVW	124	0.015 (-0.062, 0.091)	0.71	-0.001	0.002	0.45	314.17	123	1.10e-18
	Weighted median		-0.033 (-0.113, 0.048)	0.43						
	Weighted mode		-0.092 (-0.275, 0.090)	0.32						
	MR-Egger		0.136 (-0.187, 0.459)	0.41						
	MR-PRESSO (4 outliers)		-0.008 (-0.078, 0.061)	0.81						
Depressive symptoms	IVW	116	-0.020 (-0.140, 0.101)	0.75	-0.001	0.002	0.57	277.09	115	2.12e-05
	Weighted median		-0.093 (-0.228, 0.041)	0.17						
	Weighted mode		-0.172 (-0.508, 0.164)	0.32						
	MR-Egger		0.124 (-0.391, 0.639)	0.64						
	MR-PRESSO (3 outliers)		-0.042 (-0.152, 0.068)	0.45						
MDD	IVW	44	-0.002 (-0.046, 0.043)	0.94	-0.002	0.004	0.56	92.18	43	1.94e-05
	Weighted median		-0.019 (-0.067, 0.028)	0.43						
	Weighted mode		-0.054 (-0.146, 0.037)	0.25						
	MR-Egger		0.069 (-0.171, 0.310)	0.57						
	MR-PRESSO (2 outliers)		-0.008 (-0.047, 0.030)	0.67						
BMI	IVW	68	-0.019 (-0.071, 0.034)	0.48	-0.004	0.002	0.057	239.94	67	2.66e-21
	Weighted median		0.015 (-0.033, 0.063)	0.54						
	Weighted mode		0.026 (-0.049, 0.100)	0.50						
	MR-Egger		0.122 (-0.030, 0.274)	0.12						

	MR-PRESSO (7 outliers)		-0.035 (-0.075, 0.006)	0.10						
WHR	IVW	28	-0.050 (-0.123, 0.023)	0.18	0.002	0.004	0.57	69.96	27	1.14e-05
	Weighted median		-0.009 (-0.082, 0.063)	0.81						
	Weighted mode		-0.001 (-0.125, 0.124)	0.99						
	MR-Egger		-0.515 (-0.801, -0.228)	0.002						
	MR-PRESSO (1 outlier)		-0.013 (-0.071, 0.045)	0.67						
WC	IVW	41	0.003 (-0.052, 0.059)	0.91	-0.002	0.003	0.39	98.64	40	7.34e-07
	Weighted median		0.015 (-0.044, 0.073)	0.62						
	Weighted mode		0.065 (-0.029, 0.159)	0.18						
	MR-Egger		0.086 (-0.110, 0.283)	0.40						
	MR-PRESSO (3 outliers)		-0.019 (-0.070, 0.032)	0.48						
HC	IVW	52	-0.029 (-0.077, 0.019)	0.24	0.003	0.002	0.13	147.13	51	2.93e-11
	Weighted median		0.005 (-0.045, 0.056)	0.83						
	Weighted mode		0.043 (-0.088, 0.175)	0.52						
	MR-Egger		-0.134 (-0.277, 0.009)	0.072						
	MR-PRESSO (3 outliers)		-0.000 (-0.043, 0.043)	0.99						
BF%	IVW	378	-0.079 (-0.114, -0.044)	7.58e-06	0.000	0.001	0.57	798.36	377	2.18e-32
	Weighted median		-0.052 (-0.090, -0.013)	0.008						
	Weighted mode		-0.011 (-0.142, 0.120)	0.87						
	MR-Egger		-0.110 (-0.222, 0.002)	0.054						
	MR-PRESSO (13 outliers)		-0.073 (-0.103, -0.043)	2.55e-06						

[a] Causal estimates of changes in SDs of relative carbohydrate intake associated with each psychological well-being indicator or each 1-SD increase in each adiposity trait.

Abbreviations: BF%, body fat percentage; BMI, body mass index; CI, confidence interval; HC, hip circumference; IVW, inverse-variance weighted; MDD, major depressive disorder; MR, Mendelian randomization; OR, odds ratio; PRESSO, pleiotropy residual sum and outlier; SD, standard deviation; SE, standard error; SNP, single nucleotide polymorphism; UVMR, univariable Mendelian randomization; WC, waist circumference; WHR, waist-to-hip ratio.

Supplementary Table S11. Directional pleiotropy test and heterogeneity test for the causal association between relative carbohydrate intake and each mediator

Mediator	Directional pleiotropy test			Heterogeneity test		
	Egger intercept	SE	P value	Q statistic	Q df	P value
Positive affect	-0.001	0.008	0.90	7.44	4	0.11
Life satisfaction	-0.001	0.008	0.87	6.76	4	0.15
Neuroticism	-0.010	0.006	0.23	4.36	4	0.36
Depressive symptoms	0.000	0.007	0.96	9.87	4	0.043
MDD	-0.015	0.011	0.22	7.94	6	0.24
BMI	0.015	0.025	0.61	13.18	4	0.010
WHR	0.005	0.014	0.76	0.32	4	0.99
WC	0.007	0.015	0.66	3.52	4	0.47
HC	0.003	0.021	0.91	6.12	4	0.19
BF%	0.010	0.019	0.63	158.39	6	1.30e-31

Abbreviations: BF%, body fat percentage; BMI, body mass index; HC, hip circumference; MDD, major depressive disorder; SE, standard error; WC, waist circumference; WHR, waist-to-hip ratio.

Supplementary Table S12. UVMR estimates for the causal association between each mediator and hypertension

UVMR analysis	Method	No. of SNP	OR (95% CI)[a]	P value
**Positive affect**				
FinnGen	IVW	93	0.55 (0.40, 0.75)	1.58e-04
	Weighted median		0.60 (0.41, 0.88)	0.008
	Weighted mode		0.91 (0.39, 2.11)	0.82
	MR-Egger		0.17 (0.045, 0.63)	0.009
	MR-PRESSO (2 outliers)		0.55 (0.41, 0.74)	1.46e-05
UK Bioank	IVW	93	0.62 (0.47, 0.81)	5.47e-04
	Weighted median		0.56 (0.43, 0.74)	4.48e-05
	Weighted mode		0.40 (0.18, 0.88)	0.026
	MR-Egger		1.30 (0.43, 3.95)	0.041
	MR-PRESSO (1 outlier)		0.55 (0.44, 0.69)	1.59e-06
**Life satisfaction**				
FinnGen	IVW	68	0.56 (0.39, 0.82)	0.003
	Weighted median		0.57 (0.36, 0.89)	0.015
	Weighted mode		0.99 (0.31, 3.20)	0.99
	MR-Egger		0.067 (0.010, 0.44)	0.006
	MR-PRESSO (1 outlier)		0.53 (0.38, 0.74)	4.49e-04
UK Bioank	IVW	68	0.56 (0.41, 0.76)	1.96e-04
	Weighted median		0.42 (0.32, 0.57)	6.34e-09
	Weighted mode		0.34 (0.17, 0.71)	0.005
	MR-Egger		0.49 (0.11, 2.17)	0.35
	MR-PRESSO (4 outliers)		0.47 (0.37, 0.60)	4.37e-08
**Neuroticism**				
FinnGen	IVW	122	1.41 (1.11, 1.79)	0.006
	Weighted median		1.49 (1.13, 1.96)	0.005
	Weighted mode		0.93 (0.40, 2.16)	0.87
	MR-Egger		3.34 (1.10, 10.17)	0.035
	MR-PRESSO (3 outliers)		1.44 (1.16, 1.79)	0.001
UK Bioank	IVW	124	1.52 (1.26, 1.83)	1.66e-05
	Weighted median		1.71 (1.40, 2.08)	1.00e-07
	Weighted mode		1.93 (1.08, 3.45)	0.027
	MR-Egger		0.58 (0.27, 1.25)	0.17
	MR-PRESSO (7 outliers)		1.77 (1.53, 2.06)	1.06e-11
**Depressive symptoms**				
FinnGen	IVW	114	1.59 (1.09, 2.33)	0.017
	Weighted median		1.48 (0.94, 2.32)	0.088
	Weighted mode		1.03 (0.32, 3.38)	0.96
	MR-Egger		7.77 (1.40, 43.17)	0.021
	MR-PRESSO (4 outliers)		1.84 (1.32, 2.57)	5.08e-04
UK Bioank	IVW	116	2.20 (1.64, 2.97)	1.99e-07
	Weighted median		2.35 (1.73, 3.18)	4.10e-08
	Weighted mode		2.74 (1.23, 6.13)	0.015
	MR-Egger		2.49 (0.71, 8.77)	0.16
	MR-PRESSO (5 outliers)		2.69 (2.11, 3.44)	1.83e-12
**MDD**				
FinnGen	IVW	47	1.26 (1.09, 1.45)	0.001
	Weighted median		1.24 (1.05, 1.46)	0.010
	Weighted mode		1.19 (0.88, 1.60)	0.28

	MR-Egger		1.56 (0.67, 3.61)	0.31
	MR-PRESSO (2 outliers)		1.23 (1.08, 1.39)	0.002
UK Bioank	IVW	44	1.37 (1.23, 1.52)	1.28e-08
	Weighted median		1.26 (1.12, 1.41)	1.30e-04
	Weighted mode		1.20 (0.93, 1.55)	0.18
	MR-Egger		0.73 (0.43, 1.25)	0.26
	MR-PRESSO (2 outliers)		1..41 (1.28, 1.55)	9.73e-10
**BMI**				
FinnGen	IVW	67	1.64 (1.41, 1.90)	6.45e-11
	Weighted median		1..63 (1.38, 1.93)	1.51e-08
	Weighted mode		1.61 (1.19, 2.18)	0.003
	MR-Egger		1.21 (0.78, 1.88)	0.39
	MR-PRESSO (1 outlier)		1.72 (1.51, 1.97)	2.68e-11
UK Bioank	IVW	68	1.62 (1.42, 1.86)	2.54e-12
	Weighted median		1.59 (1.42, 1.78)	6.09e-16
	Weighted mode		1.64 (1.39, 1.93)	1.47e-07
	MR-Egger		1.12 (0.76, 1.65)	0.58
	MR-PRESSO (8 outliers)		1.60 (1.45, 1.75)	4.60e-14
**WHR**				
FinnGen	IVW	28	2.06 (1.65, 2.58)	2.07e-10
	Weighted median		2.01 (1.56, 2.58)	4.69e-08
	Weighted mode		1.72 (0.88, 3.37)	0.13
	MR-Egger		3.67 (1.35, 9.92)	0.017
	MR-PRESSO (2 outliers)		1.93 (1.60, 2.33)	2.35e-07
UK Bioank	IVW	28	1.69 (1.34, 2.14)	1.07e-05
	Weighted median		1.69 (1.39, 2.05)	9.17e-08
	Weighted mode		1.99 (1.30, 3.05)	0.004
	MR-Egger		1.19 (0.40, 3.55)	0.76
	MR-PRESSO (4 outliers)		1.63 (1.38, 1.92)	7.04e-06
**WC**				
FinnGen	IVW	40	1.66 (1.39, 1.99)	2.58e-08
	Weighted median		1.57 (1.29, 1.92)	7.87e-06
	Weighted mode		1.54 (1.09, 2.19)	0.019
	MR-Egger		1.75 (0.92, 3.31)	0.095
	MR-PRESSO (2 outliers)		1.82 (1.58, 2.10)	4.70e-10
UK Bioank	IVW	41	1.62 (1.34, 1.96)	8.02e-07
	Weighted median		1.67 (1.46, 1.92)	1.09e-13
	Weighted mode		1.69 (1.37, 2.09)	1.42e-05
	MR-Egger		1.95 (0.98, 3.86)	0.064
	MR-PRESSO (7 outliers)		1.62 (1.45, 1.81)	5.88e-10
**HC**				
FinnGen	IVW	51	1.41 (1.18, 1.68)	1.25e-04
	Weighted median		1.42 (1.18, 1.70)	1.55e-04
	Weighted mode		1.39 (1.01, 1.90)	0.048
	MR-Egger		2.50 (1.52, 4.12)	7.75e-04
	MR-PRESSO (3 outliers)		1.40 (1.18, 1.65)	2.49e-04
UK Bioank	IVW	52	1.38 (1.19, 1.60)	2.00e-05
	Weighted median		1.53 (1.35, 1.73)	1.11e-11
	Weighted mode		1.58 (1.38, 1.81)	1.75e-08
	MR-Egger		2.29 (1.49, 3.51)	3.92e-04
	MR-PRESSO (6 outliers)		1.46 (1.31, 1.63)	2.75e-08

[a] Indicates the odds for hypertension associated with each psychological well-being

indicator or each 1-SD increase in each adiposity trait.
Abbreviations: BMI, body mass index; CI, confidence interval; HC, hip circumference; IVW, inverse-variance weighted; MDD, major depressive disorder; OR, odds ratio; PRESSO, pleiotropy residual sum and outlier; SD, standard deviation; SNP, single nucleotide polymorphism; UVMR, univariable Mendelian randomization; WC, waist circumference; WHR, waist-to-hip ratio.

Supplementary Table S13. MVMR estimates for the causal association between each mediator and hypertension with adjustment for relative carbohydrate intake

Mediator	Outcome	Method	OR (95% CI)[a]	P value	No. of SNP	Instrumental validity test			Heterogeneity test		Directional pleiotropy test		
						F statistic	Q statistic	P value	Q statistic	P value	Egger intercept	SE	P value
Positive affect	FinnGen	MV-IVW	0.53 (0.39, 0.74)	1.26e-04	98	37	153.75	1.29e-04	156.52	9.46e-05	0.002	0.002	0.47
		MVMR Egger	0.54 (0.39, 0.74)	1.45e-04									
	UK Biobank	MV-IVW	0.58 (0.44, 0.77)	1.32e-04	98	37	272.22	5.22e-19	280.35	6.01e-20	0.001	0.002	0.76
		MVMR Egger	0.58 (0.44, 0.77)	1.45e-04									
Life satisfaction	FinnGen	MV-IVW	0.57 (0.39, 0.83)	0.003	74	36	120.56	2.22e-04	124.64	1.18e-04	0.002	0.002	0.47
		MVMR Egger	0.56 (0.39, 0.82)	0.003									
	UK Biobank	MV-IVW	0.55 (0.40, 0.77)	3.89e-04	74	36	221.41	2.32e-17	229.78	2.27e-18	0.001	0.002	0.57
		MVMR Egger	0.55 (0.39, 0.76)	3.65e-04									
Neuroticism	FinnGen	MV-IVW	1.43 (1.12, 1.83)	0.005	126	39	231.49	1.19e-08	234.41	8.13e-09	0.003	0.002	0.19
		MVMR Egger	1.43 (1.12, 1.84)	0.004									
	UK Biobank	MV-IVW	1.55 (1.27, 1.88)	1.21e-05	128	39	358.78	1.91e-24	377.07	7.39e-27	0.002	0.002	0.12
		MVMR Egger	1.56 (1.28, 1.89)	7.75e-06									
Depressive symptoms	FinnGen	MV-IVW	1.62 (1.10, 2.39)	0.014	118	39	210.09	1.53e-07	213.28	9.86e-08	0.002	0.005	0.97
		MVMR Egger	1.59 (109, 2.33)	0.017									
	UK Biobank	MV-IVW	2.32 (1.71, 3.13)	4.92e-08	120	39	316.14	3.10e-20	326.36	1.93e-21	0.002	0.002	0.18
		MVMR Egger	2.30 (1.70, 3.11)	5.66e-08									
MDD	FinnGen	MV-IVW	1.25 (1.07, 1.46)	0.005	51	33	94.11	7.91e-05	97.26	4.95e-05	0.005	0.003	0.23
		MVMR Egger	1.28 (1.10, 1.48)	0.001									
		MV-IVW	1.38 (1.22, 1.56)	3.36e-07	51	33	145.76	8.64e-12	152.72	1.38e-12	0.003	0.002	0.28

	UK Biobank	MVMR Egger	1.40 (1.23, 1.58)	1.80e-07									
BMI	FinnGen	MV-IVW	1.64 (1.43, 1.89)	3.48e-12	70	43	140.05	4.40e-07	146.68	1.04e-07	0.000	0.003	0.90
		MVMR Egger	1.64 (1.43, 1.90)	7.93e-12									
	UK Biobank	MV-IVW	1.58 (1.39, 1.80)	7.34e-12	71	44	298.60	1.18e-30	322.50	2.08e-34	-0.002	0.003	0.52
		MVMR Egger	1.59 (1.39, 1.82)	8.19e-12									
WHR	FinnGen	MV-IVW	1.98 (1.57, 2.50)	8.00e-09	33	29	58.26	0.001	63.59	4.99e-04	0.003	0.004	0.36
		MVMR Egger	1.95 (1.54, 2.47)	3.02e-08									
	UK Biobank	MV-IVW	1.68 (1.30, 2.16)	5.69e-05	33	28	156.40	4.87e-19	172.02	1.79e-21	0.003	0.004	0.40
		MVMR Egger	1.65 (1.28, 2.13)	1.17e-04									
WC	FinnGen	MV-IVW	1.71 (1.44, 2.03)	5.71e-10	45	37	87.64	4.66e-05	92.50	1.77e-05	0.001	0.004	0.79
		MVMR Egger	1.70 (1.42, 2.03)	5.72e-09									
	UK Biobank	MV-IVW	1.60 (1.33, 1.92)	4.90e-07	46	38	237.08	1.18e-28	255.68	1.23e-31	0.000	0.004	0.97
		MVMR Egger	1.60 (1.32, 1.94)	2.15e-06									
HC	FinnGen	MV-IVW	1.40 (1.17, 1.67)	2.15e-04	56	40	155.64	4.99e-12	161.14	1.35e-12	0.005	0.004	0.15
		MVMR Egger	1.37 (1.15, 1.63)	5.43e-04									
	UK Biobank	MV-IVW	1.38 (1.19, 1.61)	3.96e-05	57	41	287.83	1.23e-33	299.41	1.47e-35	0.004	0.003	0.24
		MVMR Egger	1.36 (1.16, 1.59)	1.24e-04									

[a] Indicates the odds for hypertension associated with each psychological well-being indicator or each 1-SD increase in each adiposity trait.
Abbreviations: BMI, body mass index; CI, confidence interval; HC, hip circumference; MDD, major depressive disorder; MV-IVW, multivariable inverse-variance weighted; MVMR Egger, multivariable Mendelian randomization Egger; OR, odds ratio; SD, standard deviation; SNP, single nucleotide polymorphism.; WC, waist circumference; WHR, waist-to-hip ratio.

Supplementary Figure S1. Flow chart of the present MR study

Abbreviations: BF%, body fat percentage; BMI, body mass index; GIANT, Genetic Investigation of ANthropometric Traits; HC, hip circumference; IV, instrumental variable; IVW, inverse-variance weighted; MDD, major depressive disorder; MR, Mendelian randomization; MVMR, multivariable Mendelian randomization; PGC, Psychiatric Genomic Consortium; PRESSO, pleiotropy residual sum and outlier; SNP, single nucleotide polymorphism; UVMR, univariable Mendelian randomization; WC, waist circumference; WHR, waist-to-hip ratio.

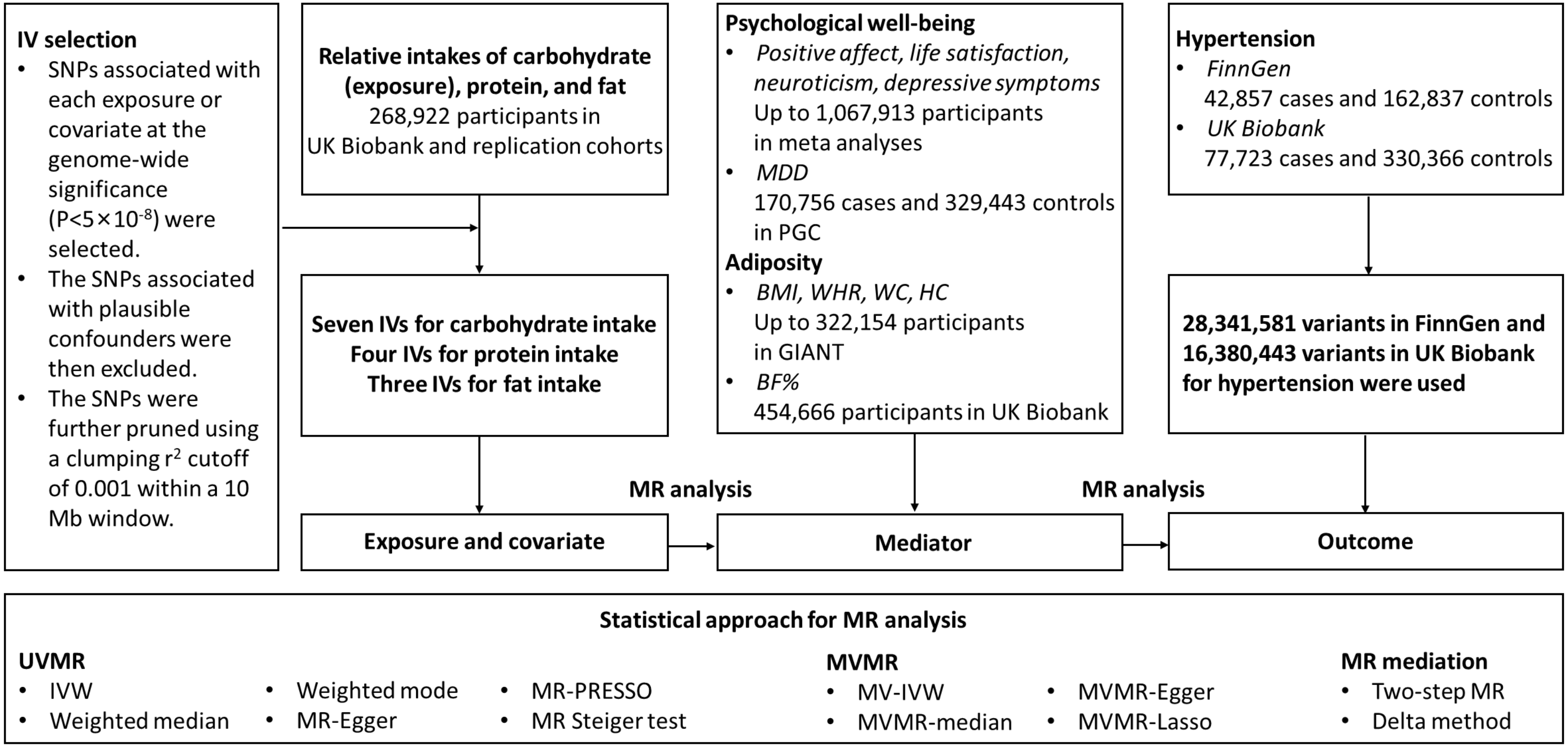

Supplement: Supplementary file 1 [file nutrients-15-04817-s001.zip › nutrients-2685074-supplementary.pdf]
